# Probing the physical limits of reliable DNA data retrieval

**DOI:** 10.1038/s41467-020-14319-8

**Published:** 2020-01-30

**Authors:** Lee Organick, Yuan-Jyue Chen, Siena Dumas Ang, Randolph Lopez, Xiaomeng Liu, Karin Strauss, Luis Ceze

**Affiliations:** 1 Paul G. Allen School of Computer Science and Engineering, University of Washington, Seattle, WA 98195 USA; 2 Microsoft, Redmond, WA 98052 USA; 3 Department of Bioengineering, University of Washington, Seattle, WA 98195 USA

**Keywords:** Molecular engineering, Computer science

## Abstract

Synthetic DNA is gaining momentum as a potential storage medium for archival data storage. In this process, digital information is translated into sequences of nucleotides and the resulting synthetic DNA strands are then stored for later retrieval. Here, we demonstrate reliable file recovery with PCR-based random access when as few as ten copies per sequence are stored, on average. This results in density of about 17 exabytes/gram, nearly two orders of magnitude greater than prior work has shown. We successfully retrieve the same data in a complex pool of over 10^10^ unique sequences per microliter with no evidence that we have begun to approach complexity limits. Finally, we also investigate the effects of file size and sequencing coverage on successful file retrieval and look for systematic DNA strand drop out. These findings substantiate the robustness and high data density of the process examined here.

## Abstract (web summary)

The physical limits and reliability of PCR-based random access of DNA encoded data is unknown. Here the authors demonstrate reliable file recovery from as few as ten copies per sequence, providing a data density limit of 17 exabytes per gram.

## Introduction

Storing digital data in synthetic DNA has become an increasingly attractive alternative to electronic archival data storage methods^1–10^. For synthetic DNA data storage to become a viable alternative to electronic archiving, many unique DNA sequences must be physically storable in a single pool and then randomly and reliably accessed. Random access requires far fewer resources to recover data since only relevant files are sequenced and analyzed. Theoretically, for maximum density, only one copy of each sequence would be necessary to perform the polymerase chain reaction (PCR) random access reaction. In practice, however, this is not the case for two reasons: stochastic variations in copy numbers that arise from sub-sampling the pool during random access, and copy number variations that arise from synthesis. Knowing the minimum copy number for each PCR reaction is crucial for storing DNA data; without it, one might store too few copies to access the data or too many, wasting orders of magnitude of density.

**Fig. 1:**
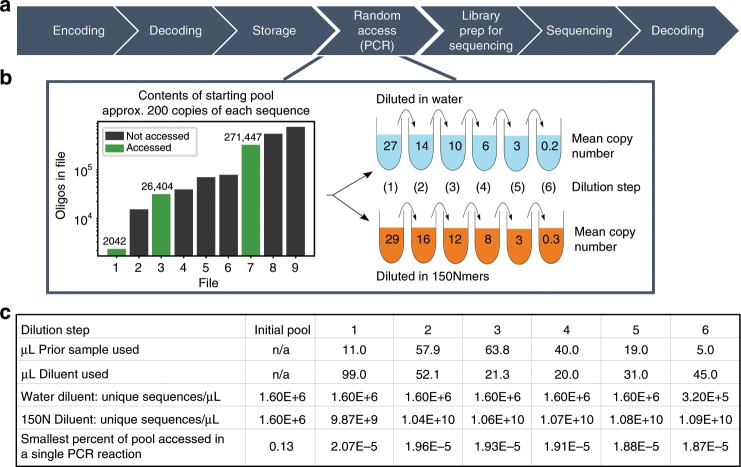
Experiment overview. **a** A high-level representation of the DNA data storage pipeline. **b** (Left) The bar chart depicts contents of the initial, undiluted pool. (Right) The illustration shows the serial nature of subsequent dilutions. Mean copy number refers to the mean number of copies of each file's unique sequences as determined by qPCR (Supplementary Note 1). One serial dilution used water as the diluent in each step; the other used a solution of 150 Nmers to dilute the pool to much greater complexity. **c** Details of how the samples were diluted. Note that the dilution steps were identical regardless of diluent. The smallest percent of pool accessed is calculated by dividing the size of the smallest file by the number of unique sequences in the 1 μL of solution used for PCR random access. This percentage refers to the 150 Nmer diluent pool since the small file in the water diluent pool is a constant 0.13%.

This paper examines the ability of PCR to recover three files, each an order of magnitude larger than the other, from truly random pools ranging from over 10^6^ to over 10^10^ unique sequences per microliter (Fig. 1). An average of 10 copies of each sequence is necessary for successful data retrieval for the three files, regardless of how many sequences per microliter are used. In addition, we also examine individual sequence behavior and find no systematic sequence loss, thus showing sequence loss is stochastic and not due to sequence design. We also look at the effects of increasing sequencing coverage to ten times greater than the coverage used in this work’s analysis and find that it is minimally effective at retrieving more sequences. This work further supports the robustness and high density storage potential of DNA, for we demonstrate we have not yet reached the limit of permissible pool complexity, and with a minimum copy number of 10 we show this process yields the densest DNA storage system to date at 17 exabytes per gram (EB g^−1^).

Previous work in this space recognized the importance of storage density for DNA to become a practical archival storage^1,3,7,10^, but the greatest complexity surrounding random access in those works reached just over 10^7^ unique sequences^10^. In more recent work, a different method of random access using bead extraction of desired strands prior to PCR random access utilized 10^18^ unique sequences^11^. However, in addition to different random access techniques and strand architecture, those methods differ substantially from the methods presented here and make it difficult to compare to this work. Notably, while in this paper random strands of DNA are encoded by 150 Nmers where each base is randomly attached during synthesis, their methods employ mutagenetic PCR on template strands which introduces an approximate 5% error rate to the final PCR product^12^ while maintaining a conserved PCR primer region. Both factors likely minimize interactions between the desired strands and the strands added for complexity. Additionally, the concentration is substantially changed in those methods depending on the complexity examined, while this work only examines random access at ~1 ng μL^−1^ concentration. Nevertheless, both works are consistent in concluding that the limit to random access complexity is not 10^10^ sequences per random access reaction. It is also important to note that PCR random access is not unique to synthetic biology, as biological research often involves using PCR in complex conditions to amplify only desired parts of a genome or gene expression data. Though the commonly analyzed human and mouse genome are both nearly 3 gigabases^13^, approximately two orders of magnitude fewer bases than examined in this work’s most complex condition, genomic libraries are often exceedingly complex because of the genome fragmentation process. While genomic analysis is also not immune to missing expected sequences (also known as dropout) and the field has developed techniques to cope with this problem, the mechanisms behind the absent sequences and the steps to mitigate it are different from more synthetic applications such as this work^14–17^. This further motivates the work presented here.

## Results

### Reducing copy number and increasing pool complexity

In this work, we randomly accessed three files from a large pool of DNA at varying copy numbers (Fig. 1b). The small file, comprised of 2042 sequences, represents ~0.1 KB of digital data. The medium file consists of 26,404 sequences and 1.7 KB of digital data, and the large file has 271,447 sequences and 18 KB.

**Fig. 2:**
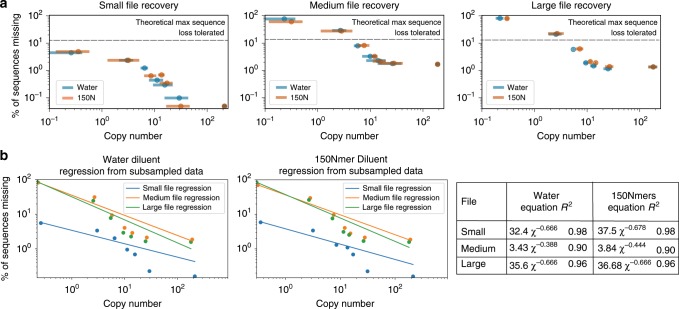
Examining sequence loss behavior. **a** Each plot illustrates a file's loss of sequences recovered at 20× coverage, directly comparing the samples diluted in water to those diluted in 150 Nmers (150nt sequences comprised of random nucleotides). The threshold of the maximum number of sequences that can be lost while still permitting file recovery is plotted for reference, as determined by previous work^10^. Error bars represent 95% confidence intervals; *x*-axis errors are taken from triplicate qPCR data (see Methods), and *y*-axis errors are the result of 100 simulations of the original sequencing data sub-sampled to 20× sequencing coverage (see Methods). **b** Each plot illustrates behavioral similarities for each file in each diluent condition, with a power regression overlayed (see Supplementary Note 4). The data used here are also sub-sampled to a sequencing coverage of 20×.

We then sequenced all three files at all stages of dilution to measure the rate of sequences lost (Fig. 2a, b). A copy number of 10, for example, means that, on average, each sequence was present 10 times in solution. The original, undiluted pool of DNA encoded nine files (1.6 M total oligonucleotides (oligos)) and was subsequently serially diluted with water to result in copy numbers ranging three orders of magnitude (Fig. 1b). To dramatically increase pool complexity, we repeated this process, this time diluting the samples with 1 ng μL^−1^ 150 Nmers, random sequences of DNA the same length as our original pool (Fig. 1c). Theoretically, we would expect to encounter a duplicate sequence only after 4^150^ (2 × 10^90^) sequences had been synthesized; thus, we would expect each strand to be unique. By diluting samples with 1 ng μL^−1^ 150 Nmers, pool complexity increased from 1.6 × 10^6^ unique sequences in the initial pool (Fig. 1b) to over 10^10^ unique sequences per microliter (see Supplementary Note 2). At ~15.6 bytes of data encoded per strand^10^, this encoding scheme and experimental protocol emulate ~150 GB of digital data per microliter, and 7 TB in the final 50 μL solution.

If PCR file retrieval fails in complex settings, we would observe a marked difference between sequences recovered in complex versus less complex settings. In addition, we might observe an inability to recover small files in complex conditions. Encouragingly, we observed neither of these symptoms. We found no distinguishable difference when comparing sequence loss between water and complex 150 Nmer dilution conditions (Fig. 2a). For the large file, only three samples distinguishably differed for the two complexity conditions; this difference was negligible, with a mean difference of sequences missing of 0.98% and standard deviation of 1.82%.

Regardless of file accessed, decreasing the copy number yielded similar behavior (Fig. 2). The loss of sequences for all three files was modeled with a power regression with *R*^2^ values ranging from 0.90 to 0.98 (Fig. 2b). However, though the medium and large files behaved almost indistinguishably, the small file did not lose sequences at a similar rate after the copy number fell below ~10 (see Fig. 2a). Instead, it lost fewer sequences than the larger two files. This is likely due to a combination of copy number being slightly higher than calculated, fewer sequences initially missing due to variation in synthesis, and the distribution of sequences being slightly more uniform (see Supplementary Note 3 for detailed analysis).

**Fig. 3:**
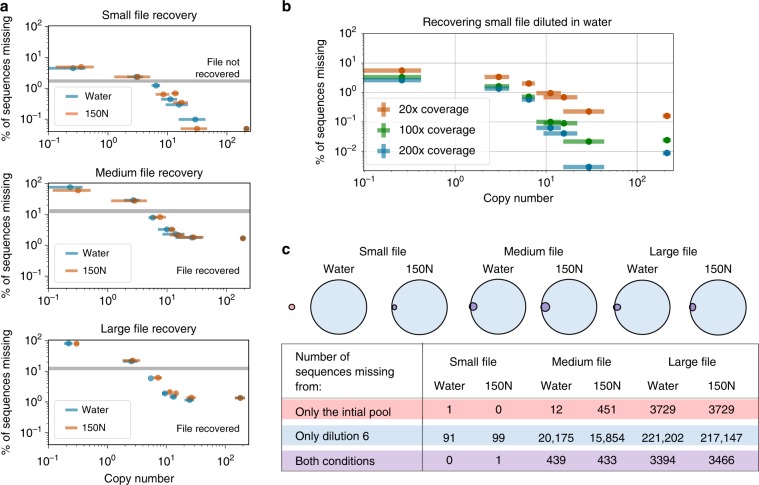
Examining file recovery, sequencing coverage, and sequence loss behavior. **a** The limit of successful, no bit error decoding is shown with the gray bar in each graph. Data points below the gray bar are samples where the file was successfully decoded and recovered with no bit errors. Done post-sequencing, decoding involves clustering sequences, finding consensus, then correcting errors^10^. A more detailed view of this data including exact copy number and sequencing coverage is in Supplementary Note 7. **b** For the small file, when each sample diluted in water was sequenced at greater depth, minimal improvement on the proportion of missing sequences occurred. The 100× coverage data were found by sub-sampling the data used to create the 200× coverage data. **c** Missing sequences are compared between the initial pool prior to any dilutions where the mean copy number was 194 (in red) and the last dilution where the mean copy number was <1 (in blue). Note the different total number of sequences in the small (2042), medium (26,404), and large (271,447) files. The fact that some sequences are missing only from the initial pool but “reappear” in the final dilution suggests that the lost sequences are a result of stochastic variation that occurs during sub-sampling for file recovery, rather than irretrievably lost due to some property of the sequence. This pattern of sequences reappearing in subsequent dilutions is shown for every dilution step in Supplementary Note 6. Note that for **a**, **b**, *x*-axis and *y*-axis error bars represent 95% confidence intervals; *x*-axis errors are taken from triplicate qPCR data (see Methods), and *y*-axis errors are the result of 100 simulations of the original sequencing data sub-sampled to 20× sequencing coverage (see Methods).

Encouragingly, the size of the file being recovered also does not impact the copy number required in complex pool conditions (Figs. 2 and 3a). Regardless of pool complexity or size of file accessed, only 10 copies of each strand on average, with a standard deviation of 3, are required for successful recovery with no bit errors. A pool complexity emulating nearly 150 GB of digital data per PCR reaction did not hinder file recovery, and the fact that recovering data from this complex pool was indistinguishable from the water-diluted pool with orders of magnitude fewer strands suggests that we have not approached the limit of pool complexity. Storing many unique sequences in one pool reduces the need for physical isolation, one of the largest density overheads facing this technology.

### Data density

Determining the need for a minimum of ~10 copies of each DNA sequence in the PCR reaction to successfully recover a file enables us to calculate a density of 17 EB g^−1^, nearly two orders of magnitude denser than prior work^7^ and closer to the maximum theoretical density predicted for DNA data storage^1^ (see Supplementary Note 5). This is due both to the wet lab techniques used (such as synthesis, sequencing, or library preparation), as well as the encoding and decoding scheme’s error tolerance (logical redundancy). In this context, physical redundancy is the number of copies of each sequence of DNA; logical redundancy in this context is the amount of extra digital information added to aid error correction and mitigate the effect of erasures (missing sequences) and it therefore increases the total number of sequences. Physical and logical redundancy are closely related regarding data density. With more logical redundancy, the system tolerates lower physical redundancy as more sequences can be lost while still allowing perfect recovery of digital data. The logical redundancy used in this work was 15%, which tolerates a maximum of 13% of sequences missing (if there are 100 sequences and we apply 15% logical redundancy, there are now 100+15 sequences and we can tolerate 15/115 missing). If the logical redundancy had been 50%, a maximum of 33% of the strands could be lost ($\frac{50}{100+50}$) (ref. ^10^). Based on the rate at which sequences are lost (Fig. 2b) and ignoring all other errors (such as insertions, deletions, substitutions), the maximum data density is 38 EB g^−1^ for the scheme with 15% logical redundancy and 124 EB g^−1^ for 50% logical redundancy. A relatively small increase in logical redundancy allows for a disproportionately lower physical redundancy with no data loss. This results in higher overall data density. It is important to note that determining the appropriate logical and physical redundancy depends on the specific DNA data storage workflow, for errors incurred by DNA synthesis, sequencing, and wet protocols used can affect sequence recovery.

### Sequence behavior

To further investigate the role of complex pools on sequence recovery, the behavior of each individual sequence was compared for both dilution conditions. We measured sequence behavior by examining the proportion of each sequence present in each dilution. Having a proportion that consistently changes for a subset of sequences would indicate that some strands are being systematically disproportionately accessed and amplified. Yet we observed no difference in sequence behavior between the two different dilution conditions. Thus, sequences may be changing proportions or are observed to be missing due to stochastic variation in sub-sampling. Further supporting this hypothesis is the fact that, although most sequences absent from the initial pool are also absent from subsequent dilutions, many of the strands “reappear” in subsequent dilutions as shown briefly in Fig. 3c; this indicates that these sequences disappeared and reappeared due to stochastic effects rather than systematic interactions that made certain sequences irretrievable. See Supplementary Note 6 for more detail and analysis.

This significant finding demonstrates the robustness of PCR itself and primer design methodology presented in prior work^10,18^. It is important to note that the encoding scheme presented in prior work^10^ and used here encodes the payload between the primer regions by using a randomized seed to generate a random payload and exclude homopolymers. While the effect of homopolymers in the payload has recently found to be minimal in practice^19^, it is encouraging to note that the care taken to randomize payloads yields no systematic sequence loss. The finding that sequences are lost stochastically coupled with the finding that there is no recovery threshold difference between samples diluted with water and those in more complex settings diluted with 150 Nmers thus assures users of this encoding scheme that we have not yet reached the limit of how complex a pool can be before it inhibits the ability to recover desired information.

### Sequencing depth

While storage density is a crucial component of DNA data storage, sequencing efficiency is also critical due to its significant time and cost. We found that the samples successfully recovered at the lowest possible copy number had a mean sequencing coverage of 35× with a standard deviation 11×, with a mean copy number of 10 and a standard deviation of 3 when accounting for all files and all diluent conditions (Supplementary Note 7, Fig. 3a). Thus, we show that it is possible to successfully recover files with no bit errors using the encoding scheme presented in prior work^10^ with a physical density that approaches the maximum theoretical density of one copy per sequence, without compromising sequencing efficiency.

We next examined the degree to which simply sequencing more of the prepared sample aids file recovery. To do so, all water-diluted samples from the small file were sequenced a second time from the exact same post-library-preparation material, this time with a much higher sequencing coverage. Previously, all samples had a mean sequencing coverage of 24.5× with a standard deviation of 1.6× and were sub-sampled randomly with replacement to 20× coverage. Here, samples had a mean sequencing coverage of 549× with a standard deviation of 148× and were sub-sampled randomly to 200× coverage. This resulted in a mean of 1.8% fewer missing sequences for 200× coverage, with a standard deviation of 1.1% (Fig. 3b). This method of increasing sequencing coverage has the benefit of not requiring additional material from the original pool because there is extra material post library preparation to sequence many times over. However, to significantly improve levels of recovery, over an order of magnitude more sequencing resources must be used. Since increasing sequencing coverage significantly increases the cost of recovery, this process is useful only as a last resort.

## Discussion

In summary, as DNA data storage becomes an increasingly viable alternative to mainstream data storage methods, the ability to perform random access on small subsets of densely stored DNA is increasingly important. With the maximum theoretical information density of 2 bits per nucleotide and and ideal copy number of 1, one could achieve a maximum theoretical density of 455 EB g^−1^ (ref. ^1^). However, there are many practical design trade offs including the ability for random access and error correction that the DNA data storage community has made. By demonstrating files with a copy number of approximately 10 can be successfully recovered, we present the most practically dense system to date at 17 EB g^−1^, nearly two orders of magnitude greater than the densest prior work^7^. Furthermore, by showing that we can reliably access files that encode 0.1 KB, 1.7 KB, and 18 KB from a complex pool emulating nearly 150 GB of digital data per PCR random access reaction, we demonstrate the ability of the storage method used in this work to enable efficient, reliable data retrieval in complex settings. We have no reason to think that we have experimentally reached the limit of how complex the pool could be for this DNA data pipeline, further supporting the robustness and unprecedented storage potential of DNA.

## Methods

### Dilution

The process of diluting the starting pool, once in water and once in 150 Nmers (strands of DNA 150 nt in length, with ‘N’ as the input when ordering from IDT to result in random sequences; see Supplementary Note 2 for a gel electrophoresis analysis) was repeated twice to confirm consistency in qPCR behavior. However, only one of the dilutions was sequenced, and those are the copy numbers reported throughout this paper. Figure 1c details the volume of diluent and sample used for each dilution step. To minimize variation in copy number due to pipetting error, the pipette used to perform the dilutions for the two samples was not adjusted between uses.

### qPCR protocol

From all dilution samples, the same three files were amplified in triplicates via qPCR. To find the most accurate standard curve for each file, an arbitrary ultramer from the relevant file was used (also in triplicate). We ordered all ultramers from IDT. See Supplementary Note 1 for primer and ultramer sequences as well as amplification efficiencies.

Each file was amplified using the following qPCR recipe: 1 μL of diluted pool, 0.5 μL of the appropriate forward primer at 10 μM, 0.5 μL of the appropriate reverse primer at 10 μM, 10 μL of 2× Kapa HiFi enzyme mix, 7 μL of molecular grade water, and 1 μL of 20× Eva Green. The following qPCR protocol was used: (1) 95 °C for 3 min, (2) 98 °C for 20 s, (3) 62 °C for 20 s, (4) 72 °C for 15 s, (5) repeat steps 2–4 as needed.

### Calculating copy numbers

For the large file, qPCR measurements were taken in triplicate, with an arbitrary ultramer from the file used as a custom qPCR standard (also measured in triplicate) to measure the number of copies of each oligo for every sample. The file’s mean amplification efficiency and difference from its standard curve are detailed in Supplementary Note 1. This identical method of qPCR with a custom standard was used to calculate the first (undiluted) copy number for the medium and small files. However, only the first, undiluted sample could be quantified in this way; this was due to the difference in amplification efficiency between the small and medium files and their respective standards, likely the result of the low number of target strands leading to non-specific amplification (see Supplementary Note 1). A different method was thus used to determine copy numbers for the remaining samples associated with the small and medium files.

For the small and medium files, the diluted samples’ copy numbers were calculated using the large file’s dilution factors. Here, because diluting the pool dilutes all three files simultaneously, the dilution factor between subsequent large file samples was the same as it was between subsequent samples for the small and medium files. Thus, the dilution factor (DF) between each subsequent dilution was found for the large file’s samples. The initial, undiluted sample’s copy number for the small or medium file (CN_0_) was then multiplied by the first (DF_1_) to yield the copy number of the first dilution:

1 $${\mathrm{{C}}}{{\mathrm{{N}}}}_{1}={\mathrm{{C}}}{{\mathrm{{N}}}}_{0}\times {\mathrm{{DF}}}.$$

This has the general formula:

2 $${\mathrm{{C}}}{{\mathrm{{N}}}}_{n}={\mathrm{{C}}}{{\mathrm{{N}}}}_{n-1}\times {\mathrm{{D}}}{{\mathrm{{F}}}}_{n}.$$

### Calculating margin of error for copy numbers

For the large file, error is presented as the 95% confidence interval found from the variation of the triplicate qPCR reactions. This was calculated from the standard error for the triplicate qPCR, translated from cycle standard error to copy number standard error using the standard curve. Next, to find the 95% CI for each copy number, the standard error was multiplied by the appropriate *t*-table value with 2 degrees of freedom (4.303).

However, for the small and medium files, calculating the 95% CI entails incorporating both the error caused by initial qPCR measurement and that from the large file’s observed dilution factors used to calculate subsequent copy numbers. This is because the equation used to calculate copy numbers is shown, where CN_*n*−1_ and DF_*n*_ both have a previously calculated 95% confidence interval:

3 $${\mathrm{{C}}}{{\mathrm{{N}}}}_{n}={\mathrm{{C}}}{{\mathrm{{N}}}}_{n-1}\times {\mathrm{{D}}}{{\mathrm{{F}}}}_{n}.$$

To calculate the qPCR measurement error for each sample (*δ*CN_*n*_), the first undiluted sample’s 95% CI was calculated in the same way as it was for the large file (detailed above). For each remaining sample, because qPCR data were not used and copy number was calculated with the large file’s dilution factor, the prior dilution sample’s copy number variation (*δ*CN_*n*−1_) was multiplied by the observed dilution factor (DF_*n*_) as measured from the large file. Thus:

4 $$\delta {\mathrm{{C}}}{{\mathrm{{N}}}}_{n}=\delta {\mathrm{{C}}}{{\mathrm{{N}}}}_{n-1}\times {\mathrm{{D}}}{{\mathrm{{F}}}}_{n}.$$

To calculate the error that results from the large file’s dilution factor measurements (*δ*DF_*n*_), we first found the greatest dilution factor that could have been calculated within the 95% confidence interval for calculated copy numbers by the following:

5 $${\mathrm{{D}}}{{\mathrm{{F}}}}_{{\mathrm{{max}}}}=\frac{{\mathrm{{C}}}{{\mathrm{{N}}}}_{n}+\delta {\mathrm{{C}}}{{\mathrm{{N}}}}_{n}}{{\mathrm{{C}}}{{\mathrm{{N}}}}_{n-1}-\delta {\mathrm{{C}}}{{\mathrm{{N}}}}_{n-1}}.$$

Thus, the variation in copy number due to the dilution factor (*δ*DF) was:

6 $$\delta {\mathrm{{D}}}{{\mathrm{{F}}}}_{n}=| {\mathrm{{C}}}{{\mathrm{{N}}}}_{n-1}\times {\mathrm{{D}}}{{\mathrm{{F}}}}_{\mathrm{max}}-{\mathrm{{C}}}{{\mathrm{{N}}}}_{n-1}\times {\mathrm{{D}}}{{\mathrm{{F}}}}_{n}|.$$

Finally, to determine the final 95% CI for small and medium files’ diluted samples’ copy numbers (*δ*CN_*n*_), the propagation equation incorporated both *δ*CN_*n*−1_ and *δ*DF_*n*_ and was represented by the following, easily solvable, standard error propagation equation:

7 $$\frac{\delta {\mathrm{{C}}}{{\mathrm{{N}}}}_{n}}{| {\mathrm{{C}}}{{\mathrm{{N}}}}_{n}| }=\sqrt{{\left(\frac{\delta {\mathrm{{C}}}{{\mathrm{{N}}}}_{n-1}}{{\mathrm{{C}}}{{\mathrm{{N}}}}_{n-1}}\right)}^{2}+{\left(\frac{\delta {\mathrm{{D}}}{{\mathrm{{F}}}}_{n-1}}{{\mathrm{{D}}}{{\mathrm{{F}}}}_{n-1}}\right)}^{2}}.$$

### Library preparation and enrichment

All files were amplified using the following recipe (primer sequences can be found in Supplementary Note 1): 1 μL of sample, 0.5 μL of the appropriate forward primer at 10 μM, 0.5 μL of the appropriate reverse primer at 10 μM, 10 μL of 2× Kapa HiFi enzyme mix, and 8 μL of molecular grade water. The following PCR protocol was used: (1) 95 °C for 3 min, (2) 98 °C for 20 s, (3) 62 °C for 20 s, (4) 72 °C for 15 s, (5) repeat steps 2–4 as needed according to prior qPCR.

Subsequent sequencing preparation via ligation was done with a modified version of Illumina TruSeq Nano ligation protocol and TruSeq ChIP Sample Preparation protocol. Step by step instructions are in Supplementary Note 8 for convenience, but briefly, samples were first converted to blunt ends with the ERP2 reagent and directions provided in the Illumina TruSeq Nano kit, then purified with AMPure XP beads according to the TruSeq ChIP protocol. An ‘A’ nucleotide was added to the 3′ ends of the blunt DNA fragments with the TruSeq Nano’s A-tailing ligase and protocol, followed by ligation to the Illumina sequencing adapters with the TruSeq Nano reagents and protocol. We then cleaned the samples with Illumina sample purification beads and enriched the sample using an 8-cycle PCR protocol given in the TruSeq Nano protocol.

For the enrichment, all samples were enriched using the following recipe: 3 μL of a ligation sample, 3 μL of the PCR Primer Cocktail provided in the TruSeq Nano kit, 12 μL of Enhanced PCR Mix provided in the TruSeq Nano kit, and 12 μL of molecular grade water. The following PCR protocol was used: (1) 95 °C for 3 min, (2) 98 °C for 20 s, (3) 60 °C for 15 s, (4) 72 °C for 30 s, (5) repeat steps 2–4 for a total of eight times. The length of enriched products was confirmed using a Qiaxcel bioanalyzer. Notably, these are the same ligation and sequencing preparation methods presented by Organick et al.^10^. Reformatted instructions are given in Supplementary Note 8 for convenience.

### Next-generation sequencing

When multiple separate samples were prepared for sequencing, these samples were mixed proportionally (e.g., a 10,000 oligonucleotide file to be sequenced with a 500,000 file would comprise 1.96% of the DNA material in this mix). The mixed sample was then prepared for sequencing by following the NextSeq System Denature and Dilute Libraries Guide. The sequencing sample was loaded into the sequencer at 1.3 pM, with a 10–20% PhiX spike-in as a control (PhiX is a reliable, adapter-ligated, well-characterized genomic DNA sample provided by Illumina).

### Sub-sampling data to calculate percent of sequences missing

To remove the effect of varying sequencing coverage, aligned sequences were randomly sub-sampled with replacement down to 20× coverage (20× coverage = number of oligos in file × 20), and the resulting number of missing strands was recorded. This was performed 100 times per file per sample to yield mean percent sequences missing and the 95% confidence interval.

### Reporting summary

Further information on research design is available in the Nature Research Reporting Summary linked to this article.

## Supplementary information

Supplementary Information

Reporting Summary

## Data Availability

The data that support the findings of this study are available from the corresponding authors upon reasonable request.

## Code availability

Code used to do the analysis presented in the paper is in Supplementary Note 4 and additional analysis code is available from the corresponding authors upon reasonable request. Encoding and decoding code is proprietary.
