# Author Correction: Probing the physical limits of reliable DNA data retrieval

**DOI:** 10.1038/s41467-020-14904-x

**Published:** 2020-02-21

**Authors:** Lee Organick, Yuan-Jyue Chen, Siena Dumas Ang, Randolph Lopez, Xiaomeng Liu, Karin Strauss, Luis Ceze

**Affiliations:** 1 Paul G. Allen School of Computer Science and Engineering, University of Washington, Seattle, WA 98195 USA; 2 Microsoft, Redmond, WA 98052 USA; 3 Department of Bioengineering, University of Washington, Seattle, WA 98195 USA

**Keywords:** Electrical and electronic engineering, Information technology

Correction to: *Nature Communications* 10.1038/s41467-020-14319-8, published online 30 January 2020.

The original version of this Article omitted the following from the Acknowledgements:

This research was supported by a sponsored research agreement and gifts from Microsoft and DARPA under the Molecular Informatics Program.

This has now been corrected in both the PDF and HTML versions of the Article.

