# Supplementary material for: Probing the physical limits of reliable DNA data retrieval
Source: Nat Commun. 2020 Jan 30;11:616. doi: 10.1038/s41467-020-14319-8 (PMC6992699; doi:10.1038/s41467-020-14319-8)
Supplement: Supplementary file 1 — Supplementary Information [file 41467_2020_14319_MOESM1_ESM.pdf]

Probing the Physical Limits of Reliable DNA Data Retrieval

Supplemental Material

Organick *et al.*

Supplementary Note 1

Each file has a unique primer pair, facilitating random access in DNA data storage.

File	Forward Primer	Reverse Primer
Small	5' ATAATTGGCTCCTGCTTGCA 3'	5' TTGCACTTTCCGCCTACATT 3'
Medium	5' AATCATGGCCTTCAAACCGT 3'	5' AACAAGACTTTCGGAGCGTT 3'
Large	5' AACATCGTGTCCAAGCAAGT 3'	5' TTGTTTGTCCACGCTTTCGA 3'

Supplementary Table 1: The forward and reverse primers needed to amplify each file.

Each file had its own ultramers to act as standards. The sequences and their percent efficiency as determined by qPCR is below. Each reaction was performed in triplicate, and to determine the standard efficiency each standard was diluted serially by an order of magnitude six times.

Ultramer	Sequence	%Amplification Efficiency
Small File Ultramer 1	ATAATTGGCTCCTGCTTGCAAGCTAGCTGCTATA CACACTCACACACGTCTCGTGTCTGCTGCTGCGA TCTGTGATAGCGTGTGAGAGACGCTAGATGCGCT GACATCTGTGCACACAGACACGAGCTAGAATGTA GGCGGAAAGTGCAA	91
Small File Ultramer 2	ATAATTGGCTCCTGCTTGCAGCTAGCTAGTGCGT GCAGCGTCTCTCAGCTCTACACTCTCTATCTACG CTAGTACGTATGTGTGACATGCGTCTGTGCAGAG CATCTACTCGCGCGAGCATACATAGCTAAATGTA GGCGGAAAGTGCAA	100
Small File Ultramer 3	ATAATTGGCTCCTGCTTGCACTAGCTAGTAGCTA CGTATGATATATACACATCAGATGCGCAGCGCGT AGCTACTATGTCACGATACTACAGCTATCGCGAT ACATATAGACGTGCACTCAGCGAGCTAGAATGTA GGCGGAAAGTGCAA	88
Small File Ultramer 4	ATAATTGGCTCCTGCTTGCATAGCTAGCGTGTCT ATCAGATGCGTGACAGTCTGTATGCGCATACATA CTGCTACTACGTACTCTACGCTATACACTAGTCT GCGACTACGAGTATGAGCAGCTCTAGCTAATGTA GGCGGAAAGTGCAA	98
Med. File Ultramer 1	AATCATGGCCTTCAAACCGTAGCTAGCTAGCGTC TACATATACAGTACTATGCAGTATATGTCATCTC AGTCAGTGTGCATCAGCTGTACAGTGCGCTACGC TACTCTATAGATAGACAGAGAGTGCATAAACGCT CCGAAAGTCTTGTT	92
Large File Ultramer 1	AACATCGTGTCCAAGCAAGTAGCTAGCTAGCTAG TCACTCGAGCGTGCACGTGCTACAGTGCGATGAC GTCTCTCTACAGATACAGTATGTATACACTATGT GCAGACGAGTCAGATATCTGCACACGAGTCGAAA GCGTGGACAAACAA	90

Supplementary Table 2: Each ultramer's sequence used for qPCR standards, as well as the resulting percent amplification efficiency of the qPCR reaction.

There are four ultramers for the small file in order to explore how much variation in amplification efficiency might be observed. A wide range of amplification efficiency results would mean it's not informative to use ultramers as quantification standards in the qPCR reactions calculating copy number. However, there was an observed 12% range, which satisfied us that picking an ultramer at random to serve as a quantification standard was sufficient. An ultramer with 110 random bases in the payload region of the strand was not ordered because it does not exclude homopolymers as our encoding scheme does, which was thought to be a potentially confounding factor.

When comparing the qPCR standard (the ultramer(s) listed above), it becomes clear that the smaller the file, the greater the difference in amplification efficiency from the standard.

Mean Percent Amplification Efficiency			
File	Standard	Water Dilution	150Nmer Dilution
Small	94%	145%	149%
Medium	92%	113%	114%
Large	90%	98%	103%

Supplementary Table 3: Comparing the percent amplification efficiency of each file's standard to its samples diluted in water and diluted in 150Nmers.

We hypothesize that due to the low number of target strands in solution, the small and medium file begin spuriously amplifying primers or other fragments of DNA in solution to create an inaccurate amplification curve. This is further supported by the fact that the small file's smallest dilution has a C_t value that completely overlaps the negative control, and also supported by the fragment analyzer results (see **S. Fig. 1**) which clearly shows the small file has more side products due to spurious amplification than the medium and large file for the same dilution.

As detailed in the Methods section of the main text, we only used qPCR data to determine the first (undiluted) copy number for the small and medium file. This is because at first, the authors tried to determine copy number based off all the qPCR results for each file. However, the amplification efficiency wasn't accurate because of the spurious amplification happening in each PCR cycle (as discussed in detail above). The resulting copy number for the last last dilutions were found to be 0.3/0.4 (diluted in water or 150Nmer respectively) for the large file, 0.7/0.6 for the medium file, and 28/29 for the small file. This would be reasonable if the small file had a much larger starting copy number than the large and medium file. However, all three have a very similar starting copy number (180, 190, and 213 for the large, medium and small file respectively). A final dilution copy number nearly two orders of magnitude higher than the other two files was clearly not accurate. In addition-the Cq value (essentially, the qPCR cycle where the Ct value was crossed) was indistinguishable from the negative control at the lowest dilution for the small file, further rendering the qPCR data unhelpful.

We therefore decided that since the large file had the best amplification efficiency when compared to its qPCR standard than the medium and small file, the most accurate way to determine copy number would be to rely on the results of the large file's qPCR. Specifically, utilizing its dilution factor. While we were able to accurately determine the medium and small file's starting copy number (pre-dilution) using custom, accurate dilution standards for each of the files, we calculated subsequent copy numbers by leaning on the qPCR data for the large file as described in the Methods.

We performed a gradient qPCR in duplicate on all dilutions and conditions for the small file. The annealing temperatures were 57, 60, 62, 65 and 67 degrees Celcius while the rest of the protocol followed those given in the Methods section of the paper. The annealing temperature used in the paper was 62C.

When compared to the paper's annealing temperature to other the other four temperatures, we find the C_t values to be either worse or indistinguishable from the 62C C_t value. A C_t value is considered worse if it took longer to amplify than the sample with an annealing temperature of 62C.

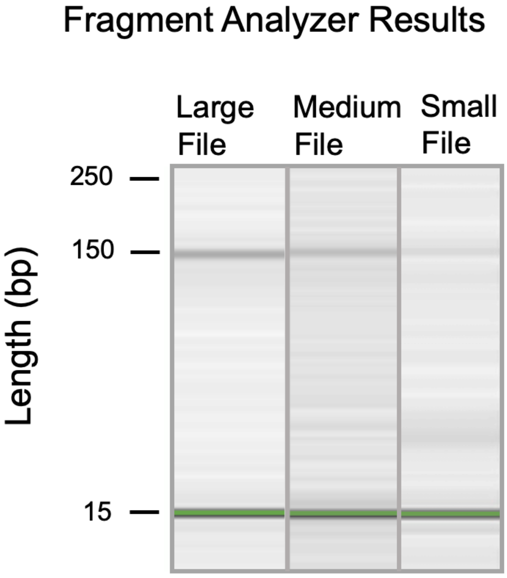

Supplementary Figure 1: Results of a fragment analyzer performed on a Qiagen QIAxcel. The desired band is at 150 bp. All samples shown here are the PCR results of the final dilution in water. The large file has the most desired product and the least spurious amplification, while the small file has the least desired product and the spurious amplification appears in such great quantity a wide band can be seen at a length of approximately 20-30bp. The medium file likely also has spurious amplification, but in such low quantities the fragment analyzer cannot detect it. All images have the same contrast and other image settings.

Of all the conditions that did not have a worse C_t value, the mean positive difference in C_t value was found to be 0.09 and the standard deviation was 0.05 cycles.

Supplementary Note 2

To ensure the 150Nmer ordered from IDT was largely the full 150nt product, gel electrophoresis was done, as shown in **S. Fig. 2**. Using FIJI analysis software, the band at the desired 150nt length was found to comprise 80% of the total product, and this was incorporated in our calculations.

The 150Nmer ordered from IDT was run through a 10% PAGE-urea gel with 1x TBE buffer and run under 200V for 25 minutes. For each sample, the Sample Buffer was the BioRad TBE-Urea Sample Buffer (Catalog 161-0768). The final volume added to each gel well was 15 μL.

The 10bp ladder was the ss10 DNA Ladder from Simplex Sciences. The final solution was 1 μL ladder, 9 μL molecular grade water, and 10 μL Sample Buffer. The 50bp ladder was from New England BioLabs, product NEB B7025. The final solution was 0.5 μL ladder, 9.5μL molecular grade water, and 10 μL Sample Buffer. To denature the ladder, this sample was denatured by incubating at 95C for 5 minutes, then immediately transferred into the gel well. The 150Nmer sample contained 5 μL of 1 ng/μL 150Nmer sample as measured by a ssDNA standards with a Qubit 3.0 Fluorometer. 5 μL 1x TE buffer was added, along with 10 μL Sample Buffer.

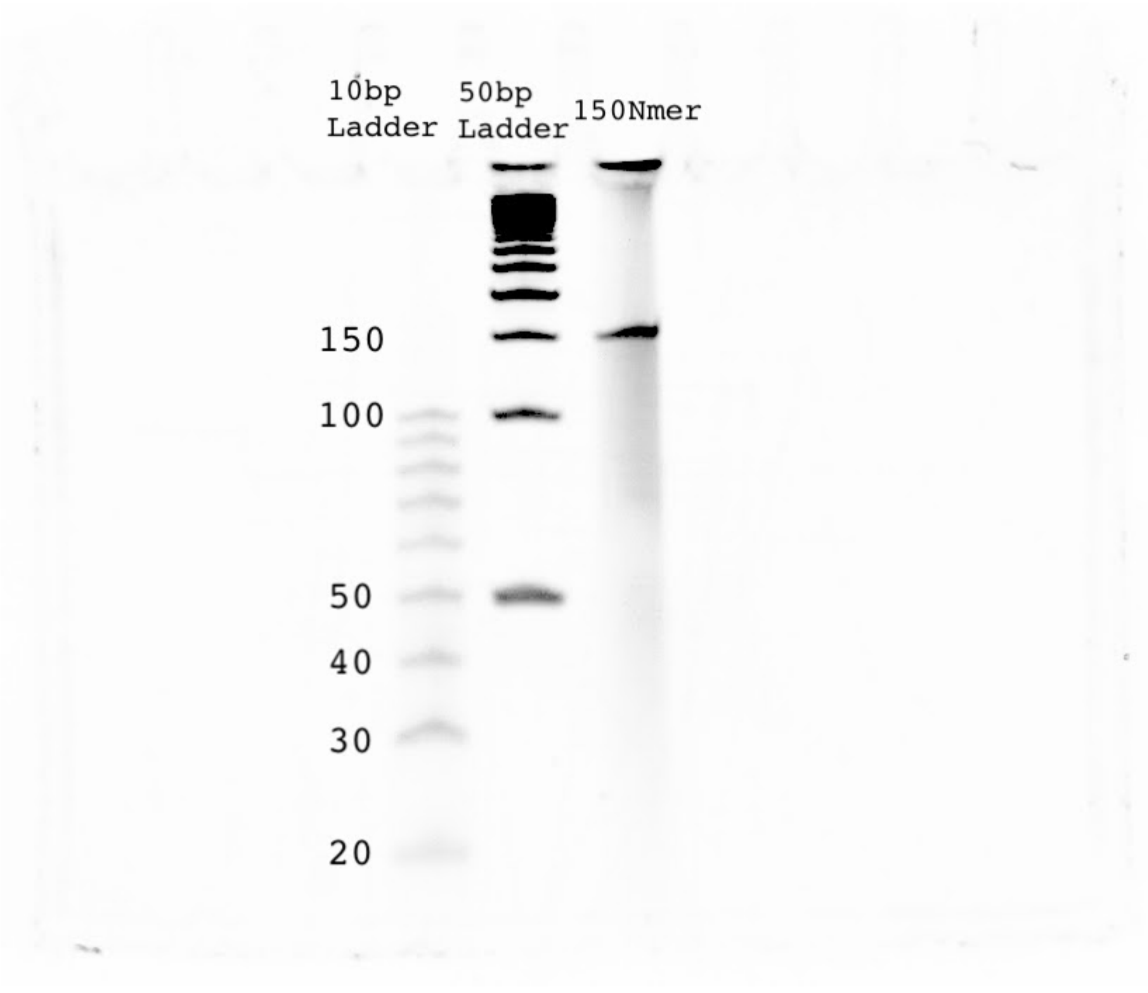

Supplementary Figure 2: Gel electrophoresis of the 150Nmer. Please note that the residue left behind in the well for both the 50bp ladder and the 150Nmer is not thought to be DNA product, as it did not migrate.

To determine how complex the pool diluted in 150Nmers actually is, we must find out how many strands there are per microliter. We can then do simple arithmetic to calculate the number of sequences per microliter (the starting sample of each PCR reaction).

Using the DNA Copy Number Calculator provided by ThermoFisher, we can input that the 150Nmers are 325 (g/mol)/bp because it is single stranded, and that it is a custom DNA fragment 150 nucleotides in length. With this information, we can now see that there are ideally $1.2 * 10^{10}$ strands of DNA per nanogram, but in reality due to imperfect synthesis we multiply this by 80% to determine a more accurate number of full length strands per nanogram. The result is $0.96 * 10^9$ full length strands of DNA per nanogram.

Due to the fact that each step of the dilution protocol adds a specified amount of 1ng/μL 150Nmer solution, it is simple to multiply that quantity by $0.96 * 10^9$ and divide by the total resulting volume to get the number of unique strands per μL being added to the previous unique strands per μL.

The amount of digital data these strands emulate is found by knowing that 200.2MB are encoded in 13,448,372 unique DNA sequences (from Organick et al. 2018), so each unique DNA sequence contains about 15.6 bytes. To calculate the total amount of data, we multiply this number by the number of different DNA sequences in solution.

The exact numbers are shown below:

*g/mol/bp**: 325

Fragment length (nt): 150

Strands/ng: 9,882,256,410

Bytes/MB: 1024^2

GB/μL: 143.6

TB/45μL: 6.310

TB in Final 50μL Solution: 7.002

**Note that these strands were ssDNA.*

Supplementary Note 3

As seen in **Fig. 2** and **Fig. 3**, it appears that the small file does not lose sequences at the same rate as the other two files. Upon further investigation, this is likely due to a combination of factors. First, the copy number is likely slightly higher than what was calculated, thus explaining the lack of sequences lost. Second, there are fewer sequences initially missing from the file. Third, as a byproduct of synthesis, the distribution of sequences is slightly more uniform (**S. Fig. 3**), further increasing the likelihood of greater sequence recovery.

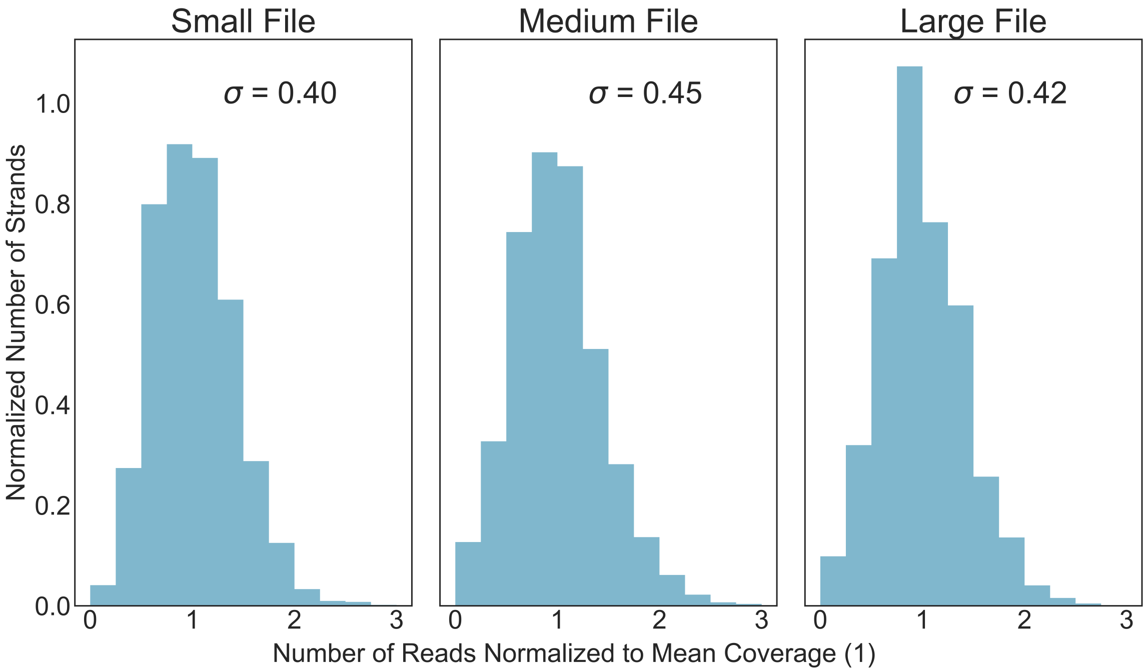

Supplementary Figure 3: Comparisons of normalized initial sequence distributions prior to dilution (with a mean copy number of 194) with standard deviation from the mean overlayed.

One hypothesis was that poor alignment scores were incorrectly identifying sequences for the small file, therefore giving the appearance of fewer sequences missing. However, as shown in **S. Table 4**, this is not supported.

File	Water	150Nmers
Small	130 ± 38	68 ± 71
Medium	29 ± 56	31 ± 58
Large	140 ± 26	140 ± 24

Supplementary Table 4: Alignment scores (mean ± standard deviation) for each file and diluent at the last dilution step.

Another hypothesis was that many sequencing reads were in fact aligning to more than one reference sequence, resulting in what's known as a chimera alignment. A chimera alignment is when one read is incorrectly identified as matching two or more reference sequences. However, as shown in **S. Table 5**, we did not find the small file to have significantly fewer reads with only one alignment.

File	Water	150Nmers
Small	97	99
Medium	99	99
Large	97	98

Supplementary Table 5: Percent of reads with one alignment for each file and diluent at the last dilution step.

To ensure that chimera alignments weren't causing false sequence recovery rates, we then examined the number of alignments per chimera read. If the small file had many more alignments per chimera read, that could explain the observed sequence recovery rates. However,as shown in **S. Table 6**, there was virtually no difference between files.

File	Water	150Nmers
Small	2.1 ± 0.25	2.0 ± 0.21
Medium	2.0 ± 0.17	2.0 ± 0.16
Large	2.1 ± 0.30	2.1 ± 0.23

Supplementary Table 6: Number of alignments (mean $\pm$ standard deviation) per chimera read for each file and diluent at the last dilution step.

For discussion of the decoding behavior of the small file, please see **Supplementary Note 7**.

Supplementary Note 4

Power regressions for fitting curves in **Fig.3** were found in Python. The following is the code used to generate both the line of best fit and the associated R^2 values:

```
# Python 2.7 was used
from scipy.optimize import curve_fit

def power(x, a, b):
    y = a * x ** -b
    return y

def get_regressions(xdata, ydata):
    """
    Given xdata (a list of copy numbers) and ydata (a list of the
    percentage of file missing), prints the resulting power line
    of best fit and associated R^2 value
    """
    popt, pcov = curve_fit(power, xdata, ydata)
    # popt[0] is the coefficient of x given by the function "power" (a)
    # popt[1] is the exponent of x given by the function "power" (b)
    x_linspace = np.linspace(min(xdata), max(xdata), 50)
    power_y = popt[0]*x_linspace**-popt[1]


    # Manually Calculating R^2 value
    residuals = ydata - power(xdata, popt[0], popt[1])
    ss_res = np.sum(residuals**2)
    ss_tot = np.sum((ydata - np.mean(ydata))**2)
    rsq = 1 - (ss_res / ss_tot)

    power_equation = 'y = ' + str(popt[0]) + r" * x **-" + str(popt[1])

    print "The line of best fit is", power_equation
    print "The R^2 value is", rsq
```

Supplementary Note 5

Using the DNA Copy Number Calculator provided by ThermoFisher, we can input that the 150Nmers are 325 (g/mol)/bp because it is single stranded, and that it is a custom DNA fragment 150 nucleotides in length. With this information, we can now see that there are $1.2 * 10^{10}$ strands of DNA per nanogram.

By knowing 15.6 bytes are encoded in each strand (Organick et al. 2018), we can then do simple arithmetic to determine the maximum amount of data that can be stored and retrieved using the encoding scheme also used in Organick et al. 2018.

The exact numbers are shown below:

*g/mol/bp**: 325

Fragment length (nt): 150

Strands/ng: 12,352,820,513

Bytes/strand: 15.6

Minimum copy number: 10

$$\frac{Bytes}{ng} = \frac{\frac{strands}{ng}}{minimum\ copy\ number} * 15.6 \frac{Bytes}{strand} = 19,270,400,000$$

$$\frac{EB}{g} = \frac{\frac{Bytes}{ng}}{1024^6 \frac{Bytes}{EB}} * 10^9 \frac{ng}{g} = 16.7$$

* Note DNA here is single stranded.

Supplementary Note 6

To investigate the role of complex pools on sequence recovery, the behavior of each individual sequence was compared between dilution conditions to check for systematic trends in recovery.

For example, each sequence in the third water dilution was compared to its same sequence in the third 150Nmer dilution. First, each file was normalized by the population fraction equation:

$$\tilde{C} = \frac{C}{T}$$

Here, $\tilde{C}$ is the normalized coverage for a given strand, C is the raw number of times the strand was seen by the sequencer and T is the total number of reads the sequencer read for that sample.

The difference in recovery behavior between the two identical sequences was then found by taking the difference between $\tilde{C}$ values for each sequence (**S. Fig. 4, S. Fig. 5, S. Fig. 6**). A skewed recovery might show sequences behaving in a systematic way, either failing to appear in the water diluted sample (negative values) or appearing much more in the water diluted sample (positive values). However, this was not found to be the case, as all samples were found to have a mode of 0 and clustered heavily around 0 largely symmetrically, showing that most sequences do not have much change in frequency regarding pool complexity.

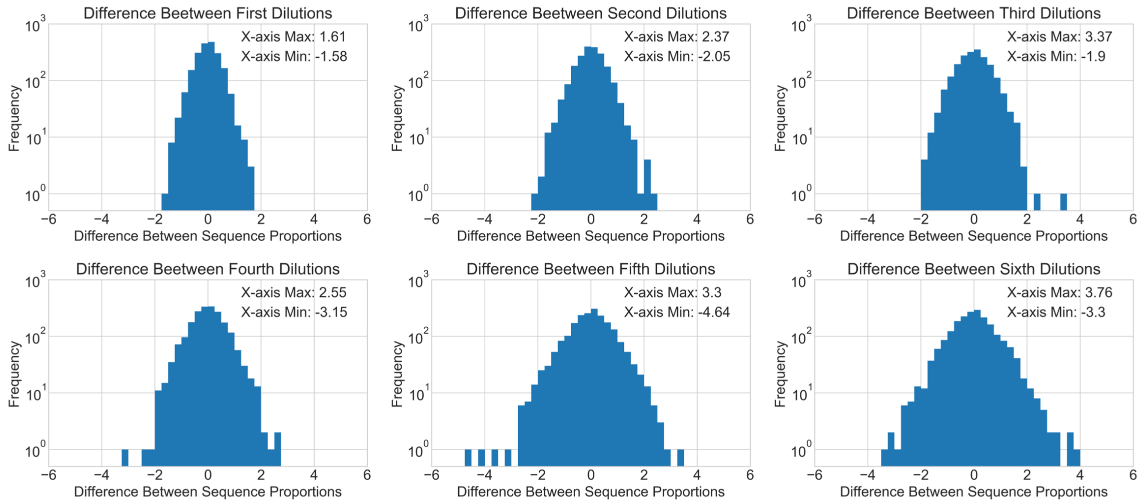

Supplementary Figure 4: All comparisons between dilution conditions for the small file.

Some of the variation in normalized coverage may have come from stochastic variation as a product of subsampling the pool at the dilution step, and at the PCR retrieval step. This is supported by **S. Fig. 7**, in which Venn diagrams compare the lost sequences from one dilution to the next. In **S. Fig. 7**, it is interesting to note that the lost sequences in subsequent dilutions are not merely supersets of those from the prior dilution, despite them being serially diluted. However, when the initial undiluted sample's missing strands are compared to the final dilution's missing

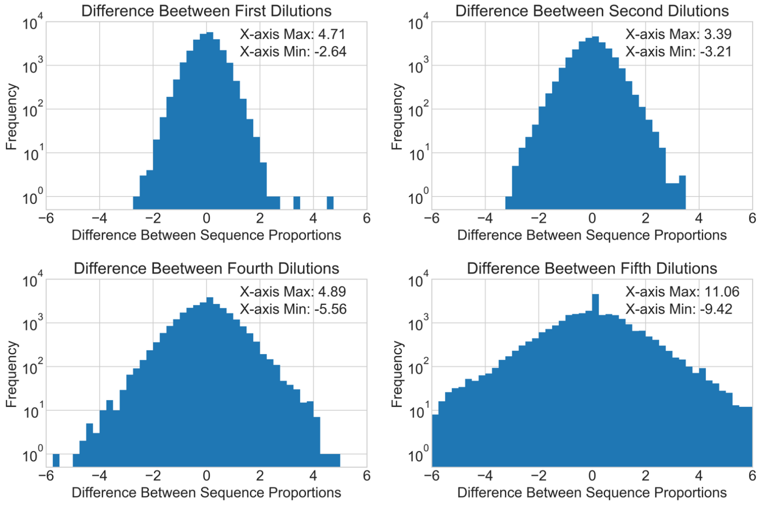

Supplementary Figure 5: All comparisons between dilution conditions for the medium file.

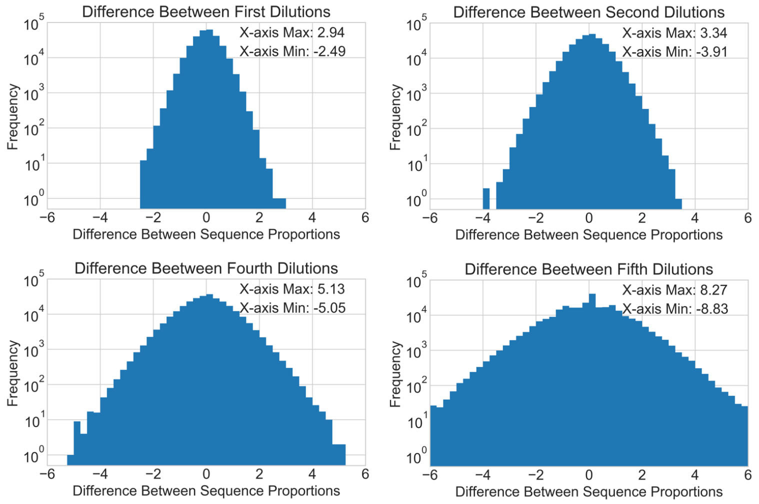

Supplementary Figure 6: All comparisons between dilution conditions for the large file.

strands (with copy numbers 194 vs 0.3 respectively), we see that the missing strands from the final dilution are still not a perfect superset. This illustrates the stochastic nature of sampling sequences, and does not indicate a strong, underlying property of strands that makes them unrecoverable.

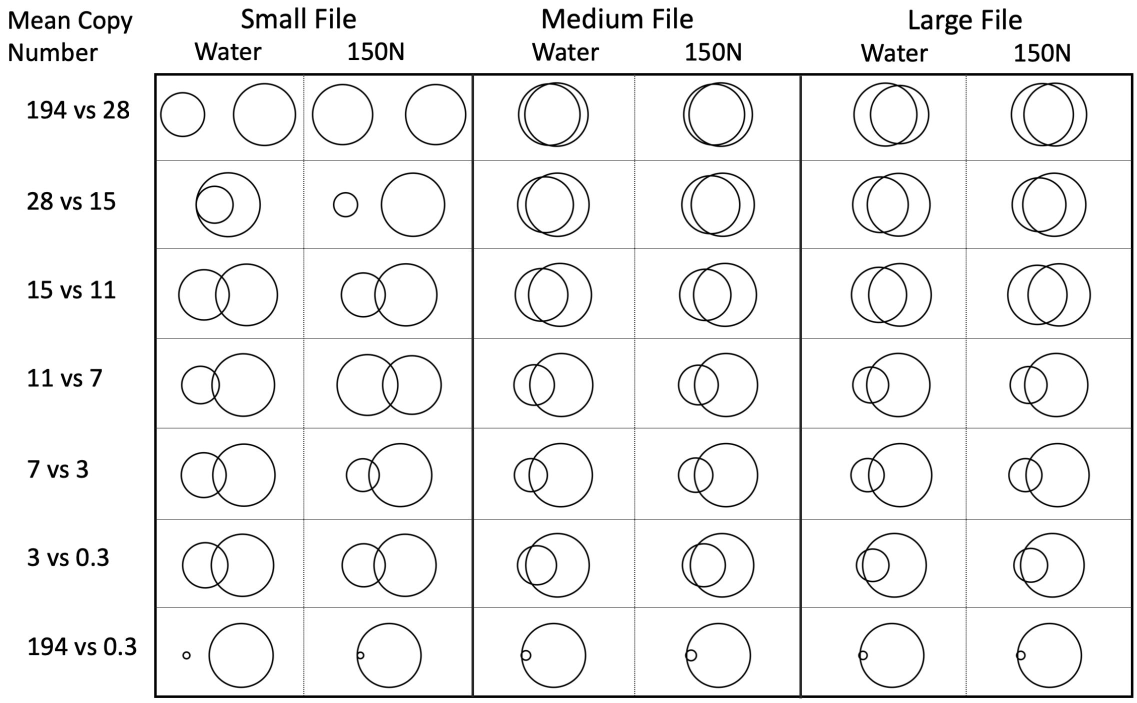

Supplementary Figure 7: For each file and diluent condition, a Venn diagram compares the set of sequences missing from the two copy numbers compared (given in the leftmost column). Each circle represents the set of sequences that were not recovered, and the overlap between the circles represents the sequences missing from both dilutions.

Supplementary Note 7

As shown in **Fig. 3a**, there is a threshold at which the data can no longer be decoded with no bit errors. The following provides a more granular presentation of that data for the small file (**S. Table 7**), medium file(**S. Table 8**), and large file (**S. Table 9**). Sequencing coverage is determined by comparing alignment of actual sequences recovered to sequences expected by the reference template; it is the result of total alignments divided by sequences in the file. Decoding is blind to any references and is thus more vulnerable to losing sequences.

Diluent	Copy Number	Sequencing Coverage	Decoded?
Water	11.2	22.5	Yes
Water	6.5	25.5	Yes
Water	3.0	25.0	No
Water	0.3	21.9	No
150Nmers	13.6	20.5	Yes
150Nmers	8.5	27.9	Yes
150Nmers	3.2	20.5	No
150Nmers	0.4	20.8	No

Supplementary Table 7: Sequencing and recovery data for the small file.

Diluent	Copy Number	Sequencing Coverage	Decoded?
Water	14.0	22.0	Yes
Water	10.0	30.7	Yes
Water	5.8	23.6	No
Water	2.7	23.3	No
150Nmers	28.1	21.8	Yes
150Nmers	15.4	29.0	Yes
150Nmers	12.1	22.6	No
150Nmers	7.6	23.7	No

Supplementary Table 8: Sequencing and recovery data for the medium file.

Diluent	Copy Number	Sequencing Coverage	Decoded?
Water	180.2	22.2	Yes
Water	9.5	54.5	Yes
Water	5.5	33.5	No
Water	2.6	29.0	No
150Nmers	14.6	23.1	Yes
150Nmers	11.5	42.5	Yes
150Nmers	7.2	25.6	No
150Nmers	2.7	37.9	No

Supplementary Table 9: Sequencing and recovery data for the large file.

Note that as shown in **Fig. 3a** the small file fails to decode with a lower percent of sequences missing than the medium or the large file. However, the ultimate difference is not as great as it might

seem as the copy numbers are different for each data point, and in fact the small file successfully decodes with the average lowest copy number of 7.5 (when averaging both water and 150Nmer diluent conditions), while the medium and large file are 12.7 and 10.5 respectively. This data is presented in **S. Tables 7**, **8**, and **9**.

Furthermore, we have performed an in-depth analysis of the decoding process for all three files and did not see significant deviations in other error metrics such as global rate of errors or rate of insertion, deletion, or substitution errors per coordinate. The difference in the observed behavior likely comes from the small file size of 2,042 sequences, for at small sizes random effects of noise become more pronounced and larger deviations become more likely. Philosophically, this is similar to the law of large numbers. Behavior is more predictable when a larger number a random variables are summed together.

Supplementary Note 8

The ligation protocol used is a combination of the Illumina TruSeq ChIP Sample Preparation protocol and the Illumina TruSeq Nano Library Prep protocol, using the Illumina TruSeq Nano reagents. The following are the step-by-step directions used in this work.

1. Add 40 μL ERP2 to each well with 60 μL of PCR product
2. Pipette up and down to mix
3. Place on the thermal cycler and run the ERP program (30 min at 30C) then place on ice. Each well contains 100 μL.
4. Add 160 μL well-mixed AMPure XP Beads to each well of the PCR plate containing 100 μL End Repair Mix.
5. Gently pipette the entire volume up and down 10 times to mix thoroughly.
6. Incubate the PCR plate at room temperature for 15 minutes.
7. Place the PCR plate on a magnetic stand at room temperature for 15 minutes or until the liquid is clear.
8. Using a 200 μL single channel or multichannel pipette set to 127.5 μL, remove and discard 127.5 μL of the supernatant from each well of the PCR plate.
9. Repeat step 8 one time.

   NOTE- Leave the PCR plate on the magnetic stand while performing the following 80% EtOH wash steps (10-12).
10. With the PCR plate on the magnetic stand, add 200 μL freshly prepared 80% EtOH to each well without disturbing the beads.
11. Incubate the PCR plate at room temperature for 30 seconds, and then remove and discard all of the supernatant from each well. Take care not to disturb the beads.
12. Repeat steps 10 and 11 one time for a total of two 80% EtOH washes.
13. Let the PCR plate stand at room temperature for 15 minutes to dry, and then remove the plate from the magnetic stand.
14. Resuspend the dried pellet in each well with 17.5 μL Resuspension Buffer (RSB). Gently pipette the entire volume up and down 10 times to mix thoroughly.
15. Incubate the PCR plate at room temperature for 2 minutes.
16. Place the PCR plate on the magnetic stand at room temperature for 5 minutes or until the liquid is clear.
17. Transfer 15 μL of the clear supernatant from each well of the PCR plate to the corresponding well of a new 96-well 0.3 ml PCR plate.
18. Add 15 μL of A Tailing Ligase (ATL) to the 15 μL of supernatant from previous step.
19. Briefly spin down on a standard, small benchtop centrifuge.
20. Place on the thermal cycler and run the ATAIL70 program listed below. Each well contains 30 μL.

(a) Choose the preheat lid option and set to 100C
    i. 37C for 30 minutes
    ii. 70C for 5 minutes
    iii. 4C for 5 minutes

21. Add the following reagents from the Illumina TruSeq Nano ligation kit IN ORDER:
    (a) RSB (2.5 μL)
    (b) LIG2 (2.5 μL)
    (c) DNA adapter (2.5 μL)

22. Pipette up and down, centrifuge briefly

23. Run lig program listed here in step a.
    (a) Choose the preheat lid option and set to 100C
        i. 30C for 10 minutes
        ii. Hold at 4C
    (b) Add 5 μL STL to each well, and then mix w/pipette. NOTE- You can hold this mixture at -20C overnight with no trouble.

24. Vortex Sample Purification Beads (SPB) until well dispersed.

25. Perform steps a through l using the Round 1 volumes.
    (a) Add SPB to each well, and then mix thoroughly as follows.
        i.

Round 1	42.5 μL
Round 2	50 μL

    (b) Pipette up and down.
    (c) Incubate at room temperature for 5 minutes.
    (d) Place on a magnetic stand and wait until the liquid is clear (2–5 minutes).
    (e) Remove and discard all supernatant from each well.
    (f) Wash 2 times as follows.
        i. Add 200 μL freshly prepared 80% EtOH to each well.
        ii. Incubate on the magnetic stand for 30 seconds.
        iii. Remove and discard all supernatant from each well.
    (g) Use a 20 μL pipette to remove residual EtOH from each well.
    (h) Air-dry on the magnetic stand for 5 minutes.
    (i) Add RSB to each well.
        i.

Round 1	52.5 μL
Round 2	27.5 μL

    (j) Remove from the magnetic stand, and then mix thoroughly as follows. Pipette up and down.
    (k) Incubate at room temperature for 2 minutes.
    (l) Place on a magnetic stand and wait until the liquid is clear (2–5 minutes).
    (m) CONTINUE TO STEP 26

26. ROUND1- Transfer 50 μL supernatant to the corresponding well of the CAP plate.
27. Repeat steps a through l with the new plate using the Round 2 volumes.
28. ROUND2- Transfer 25 μL supernatant to the corresponding well of the PCR plate.

SAFE STOPPING POINT If you are stopping, seal the plate and store at -25C to -15C for up to 7 days.

At this point, ligation and purification is done.

The following is the recipe for each ligated sample:

Sample	Concentration	Volume (μL)
DNA mix		3
PPC (PCR Primer Cocktail)	10x	3
EPM (Enhanced PCR Mix)	2.5x	12
Molecular Grade Water		12
Total		30

Follow the following thermocycling protocol with the above 30 μL mixture:

- Choose the preheat lid option and set to 100C
- 95C for 3 minutes
- 8 total cycles of:
  - 98C for 20 seconds
  - 60C for 15 seconds
  - 72C for 30 seconds
- 72C for 3 min
- Hold at 4C
